# Physiological and pharmacokinetic effects of oral 1,3-dimethylamylamine administration in men

**DOI:** 10.1186/2050-6511-14-52

**Published:** 2013-10-04

**Authors:** Brian K Schilling, Kelley G Hammond, Richard J Bloomer, Chaela S Presley, Charles R Yates

**Affiliations:** 1 Department of Health and Sport Sciences, The University of Memphis, 161 Roane Fieldhouse, 38152 Memphis, TN, USA; 2 University of Tennessee Health Sciences Center, Memphis, TN, USA

**Keywords:** 1,3-dimethylamylamine, Pharmacokinetics, Dietary supplements

## Abstract

**Background:**

1,3-dimethylamylamine (DMAA) has been a component of dietary supplements and is also used within "party pills," often in conjunction with alcohol and other drugs. Ingestion of higher than recommended doses results in untoward effects including cerebral hemorrhage. To our knowledge, no studies have been conducted to determine both the pharmacokinetic profile and physiologic responses of DMAA.

**Methods:**

Eight men reported to the lab in the morning following an overnight fast and received a single 25 mg oral dose of DMAA. Blood samples were collected before and through 24 hours post-DMAA ingestion and analyzed for plasma DMAA concentration using high-performance liquid chromatography–mass spectrometry. Resting heart rate, blood pressure, and body temperature was also measured.

**Results:**

One subject was excluded from the data analysis due to abnormal DMAA levels. Analysis of the remaining seven participants showed DMAA had an oral clearance of 20.02 ± 5 L∙hr^-1^, an oral volume of distribution of 236 ± 38 L, and terminal half-life of 8.45 ± 1.9 hr. Lag time, the delay in appearance of DMAA in the circulation following extravascular administration, varied among participants but averaged approximately 8 minutes (0.14 ± 0.13 hr). The peak DMAA concentration for all subjects was observed within 3–5 hours following ingestion and was very similar across subjects, with a mean of ~70 ng∙mL^-1^. Heart rate, blood pressure, and body temperature were largely unaffected by DMAA treatment.

**Conclusions:**

These are the first data to characterize the oral pharmacokinetic profile of DMAA. These findings indicate a consistent pattern of increase across subjects with regards to peak DMAA concentration, with peak values approximately 15–30 times lower than those reported in case studies linking DMAA intake with adverse events. Finally, a single 25 mg dose of DMAA does not meaningfully impact resting heart rate, blood pressure, or body temperature.

**Trial registration:**

NCT01765933

## Background

The stimulant 1,3-dimethylamylamine (DMAA; also known as methylhexaneamine) had been a component of many dietary supplements in the United States until the Food and Drug Administration warned retailers that DMAA did not have ample evidence of safety [1]. Little is known about the effects of oral administration of this compound in humans, but animal studies have indicated that the LD_50_ is 39 mg∙kg^-1^ for intravenous [2] and 185 mg∙kg^-1^ for intraperitoneal [3] administration. Dietary supplements containing DMAA were once widely available, with an estimated 440,000,000 servings of such supplements sold in recent years [4]. These doses are primarily as a component of “pre-workout” supplements marketed at those who exercise. The safety of this simple aliphatic amine has been called into question recently, partially based on case reports documented in New Zealand suggesting adverse outcomes following oral DMAA ingestion [5,6]. In these case studies, which cite cerebral hemorrhage following DMAA ingestion, individuals reported ingesting a single dose of DMAA (for its stimulant properties), often in conjunction with caffeine and alcohol [5,6]. Contrary to these case studies, several prospective investigations to date using recommended doses have not shown any untoward side effects [7-12]. Despite this, DMAA has been banned in many countries, including the United States. The purpose of this study was to characterize the plasma concentration profile and associated physiological effects following a single 25 mg oral dose of DMAA.

Previously, Gee and coworkers [6] reported a patient that purportedly ingested two “tablets” containing DMAA (later confirmed by analysis to contain 278 mg of DMAA per “capsule”: total dosage = 556 mg), along with 150 mg of caffeine and one can of beer. In a subsequent report [5], biochemical analysis of blood samples obtained from patients ingesting a 12.5 and 132 mg dose of DMAA indicated plasma DMAA concentrations of 760 ng∙mL^-1^ (17 hours post-ingestion) and 1090 ng∙mL^-1^(1.66 hours post-ingestion). A third patient was noted to have a plasma DMAA concentration of 2310 ng∙mL^-1^ (2 hours post-ingestion); however, no information was provided regarding the ingested dosage of DMAA. As indicated in these papers, it should be noted that other chemicals may have been taken along with the DMAA-containing products (*e*.*g*., alcohol, caffeine, phenethylamine, and cannabis).

As mentioned previously, DMAA is often ingested with caffeine for a proposed combined effect leading to greater arousal than either alone. Four studies are available that used this combination in a placebo-controlled design, and the health implications of DMAA in these studies has been unremarkable. In varying concentrations of caffeine and DMAA, there appears to be no effect on heart rate [8], while DMAA affected blood pressure and the rate-pressure product in a dose-dependent manner. These changes were not related to changes in norepinephrine or epinephrine, and caffeine/DMAA does not appear to be additive. Ergogenic effects of the combination of these substances on running performance have not yet been supported [7].

Whitehead *et al*. [12] assigned men to a placebo (n = 13) or a proprietary-blend supplement (Jack3d®;n = 12) condition, where subjects were directed to consume their assigned supplement on training days for the course of 10 weeks. No condition differences were noted for blood pressure, heart rate, or any variable of blood borne markers of health. Another sample of men (n = 7) consumed Jack3d®, while men (n = 4) and women (n = 2) consumed a different proprietary-blend supplement (OxyELITE Pro®) for two weeks [9]. In this open-label design, no significant chronic changes in heart rate, blood pressure, or rate pressure product noted for either product, although OxyELITE Pro® acutely increased systolic blood pressure. Since both of these products contain unique additional substances, it is difficult to ascribe responses to DMAA.

Finally, two studies examined OxyELITE Pro® in both acute [10] and chronic [11] conditions. Acutely, the supplement caused a significantly greater area under the curve for glycerol, free fatty acids, and kcal expenditure at rest. Heart rate, systolic blood pressure and rate pressure product were also higher with the supplement than the placebo [10]. Eight weeks of supplementation with the supplement or placebo did not demonstrate interactions for body weight, body composition, skinfold thickness, serum lipids or appetite [11].

To conclude on the safety profile of DMAA based solely on case reports would be problematic, in particular when accepting testimony from patients in uncontrolled environment, potentially under the influence of alcohol and other drugs. This is especially true in light of the fact that no prospective studies have shown these effects. Hence, the intent of the present study was to determine the pharmacokinetic profile of a single 25 mg oral dosage of DMAA alone through 24 hours post-ingestion. The results of this study, along with current available information, may provide a more comprehensive view of the effects of oral administration of this ingredient in humans.

## Methods

### Subjects and data collection

Eight healthy men (26 ± 4.1 y) were recruited to participate. Subjects met inclusion criteria by not currently smoking, and they did not have any self-reported cardiovascular or metabolic problems. Men were recruited so that there would be no possible gender effects, and since little is known about this ingredient, we thought it prudent to reduce potential confounding factors. Health history, drug and dietary supplement usage, and physical activity questionnaires were completed by all subjects and screened by an investigator to determine eligibility. Prior to participation, each subject gave written and verbal informed consent for procedures and publication of data in accordance with the procedures approved by the University of Memphis Institutional Review Board for Human Subjects Research (protocol approval number 2102). This trial is registered as NCT01765933. After giving informed consent, subjects had a dual x-ray absorptiometry (DXA) scan (Hologic QDR 4500, Bedford, MA) to measure fat mass and lean mass for descriptive purposes.

Subjects reported to the lab in the morning following an 8-hour overnight fast to minimize the possible effects of stomach contents. This is also similar to the instructions on some supplement labels. Subjects were asked to abstain from any dietary supplement containing DMAA for 72 hours prior to testing and also asked to refrain from strenuous physical activity for the 36 hours prior to testing. Following the measurement of resting heart rate (via 60 second palpation), blood pressure (standard manual procedures), and cutaneous temperature (forehead), in addition to collection of a fasting blood sample (as described below), subjects received 25 mg of DMAA in a cellulose capsule supplied by USPlabs (Dallas, TX). These are similar to capsules previously available commercially. The DMAA capsules used in this study were submitted for analysis to confirm the 25 mg dose. Mean concentration (±SD) of 10 capsules randomly selected from the same lot as the study capsules was 23.9 ± 1.9 mg. Subsequent measures of heart rate, blood pressure, and cutaneous temperature were obtained, and blood samples were taken at intervals over a 24 hour period (0.25, 0.5, 0.75, 1, 1.5, 2, 2.5, 3, 4, 5, 6, 8, 12, and 24 hr). Heart rate, blood pressure, and cutaneous temperature data were obtained to note the time effect on these variables [13]. Subjects remained in the lab for the first 8 hours of testing. They were given standardized meals for the 24- hour testing period (meals were consumed immediately after 3 and 6-hour draws, between 8 and 12-hour draws, and between 12 and 16 hours post-DMAA ingestion). They were instructed to have minimal physical activity, and return to the lab 8-hours fasted at the time of the 24-hour blood draw.

### Blood collection and processing

Peripheral IV catheters (straight, 22 ga., length: 1” polyurethane) were inserted and secured prior to first blood draw and monitored throughout the first eight hours. The IV site was immediately covered with a transparent dressing to decrease the chance of infection and to allow for catheter monitoring. Venous blood samples (approximately 5 mL) were taken from subjects at intervals as described above. The first 0.5-1 mL of each sample was discarded to avoid contamination, the sample was collected, and the catheter was flushed with 2–3 ml of 0.9% saline solution. Blood samples at 12 and 24 hr were performed with standard needle venipuncture. Following collection, blood samples were processed accordingly with sodium heparin and plasma samples were stored at −70°C until analyzed.

### Plasma DMAA analysis

**Figure 1:**
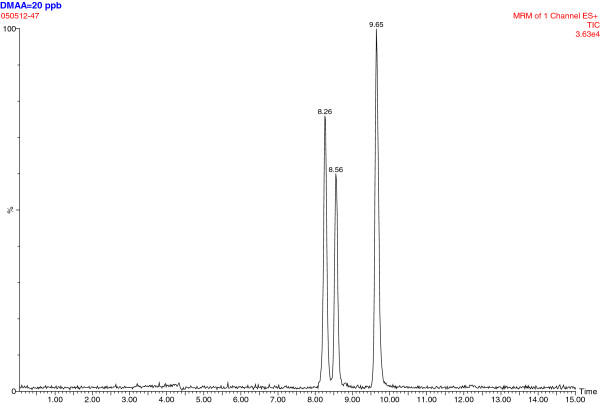
**Chromatogram of samples spiked at 20 ng/mL DMAA = 8**.**26 min and 8.56 min; 9.65 min = 2-aminoheptane.**

**Figure 2:**
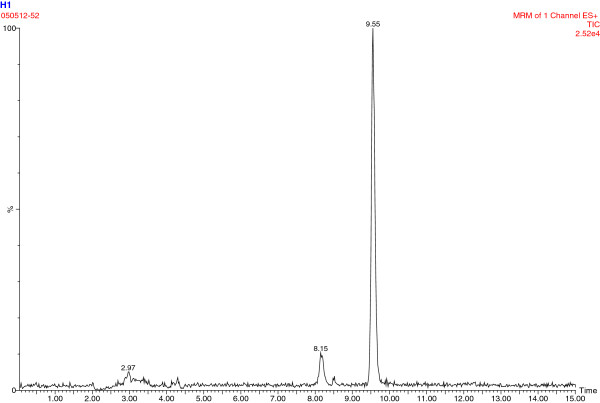
Chromatogram for subject #8 at baseline; 1.5 ng/ml DMAA.

**Figure 3:**
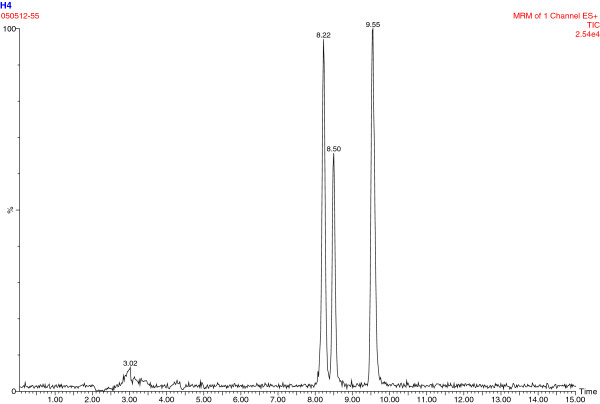
Chromatogram for subject #8 at 0.75 hrs post ingestion; 44 ng/ml DMAA.

Plasma samples from the subjects were screened with high-performance liquid chromatography–mass spectrometry (an Agilent 1200 series HPLC [Agilent Technologies, Santa Clara, CA] with an ABSciex 3200 QTrap mass spectrometer [AB-Sciex, Foster City, CA]). Based on the properties of both DMAA and human plasma, we combined TCA precipitation with LC-MS-MS using analysis of DMAA in urine as reference [14,15], and 2-aminoheptane as an internal standard. In this method, the lower limit of quantitation (LLOQ) was estimated based on the lowest level in standard curve (R^2^ > 0.99) (instrument sensitivity) and sample preparation. We forecasted that 5–50 ng/ml would cover the range of blood DMAA levels; therefore, we set up the accuracy experiment within 5–50 ng/ml-spiked levels. The LLOQ was 1–2 ng/ml (1 ng∙mL^-1^ average), and the average recovery was 92.4-97.4% when samples were spiked between 5–50 ng/ml; CV 0.9-6.8% between 5–50 ng/ml. Representative chromatograms are shown in Figures 1, 2 and 3. There is a double chromatographic peak of the racemic mixture that is made and sold commercially [14,15].

### Pharmacokinetic analysis

DMAA plasma concentration-time data were evaluated using noncompartmental analysis in Phoenix WinNonlin software with adjustment for lag time after oral administration. The area under the plasma concentration-time curve from time 0 to infinity (AUC_0-∞_) was calculated using the trapezoidal rule extrapolated to time infinity. The terminal half-life (t _1/2_) was calculated using 0.693/λz, with λz as the terminal rate elimination constant. Peak concentration (C_max_), lag time (t_lag_), time of maximum concentration (t_max_), apparent volume of distribution during the terminal elimination phase (V_z_/F), and oral clearance (CL/F) were also calculated.

### Statistical analysis

Physiological response data (heart rate, blood pressure, temperature) were analyzed using a one-way repeated measures analysis of variance. The data are presented as mean ± SD. Statistical significance was set at P ≤ 0.05, with Sidak post-hoc adjustments for multiple comparisons. Effect sizes were also calculated for selected pairwise comparisons, with corrections for correlations in repeated measures, and interpretation according to Hopkins [16].

## Results

One participant had extremely high blood levels of DMAA, including a high baseline value of 131.1 ng∙mL^-1^ and a C_max_ of 266.2 ng∙mL^-1^ occurring at 24 hours. As the participant had rising blood levels throughout the experimental window, it was impossible to calculate a terminal half-life. Upon questioning, the subject denied taking a DMAA product within 72 hours of testing, so the reason for/source of the high levels is unknown and could not be attributed to a specific methodological issue. The physiological variables for this subject were comparable to the other participants, but the subject’s DMAA t_max_ and C_max_ increased mean pharmacokinetic values by roughly 70% and 30%, respectively. Therefore, all data from the subject were excluded from the analysis. Subject characteristics for the seven subjects are presented in Table 1.

**Table 1:** Participant descriptive information (n = 7)

	**Age (y)**	**Lean body mass (kg)**	**Fat mass (kg)**	**Total body mass (kg)**	**% Fat**
Mean	26.7	68.1	11.2	79.3	13.9
SD	4.3	6.0	4.9	8.3	4.8
Range	23-36	58.7-75.8	6.1-21.4	68.7-92.8	8.2-23.1

### Physiological data

**Figure 4:**
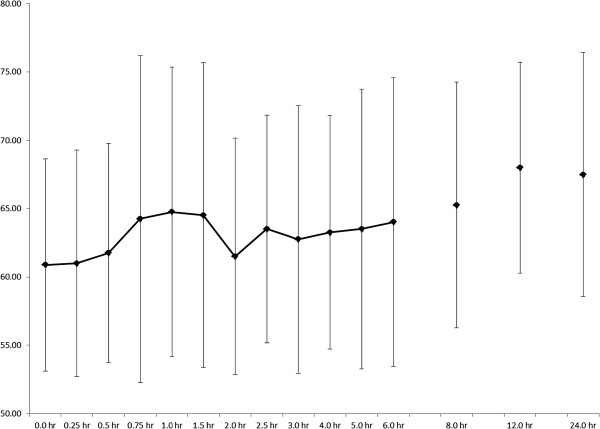
**Heart rate responses (BPM) after oral administration of 25 mg DMAA (n = 7).** Data are mean ± SD. A significant time effect was noted (p < 0.000), but no pairwise differences were detected post-hoc (p > 0.05).

**Figure 5:**
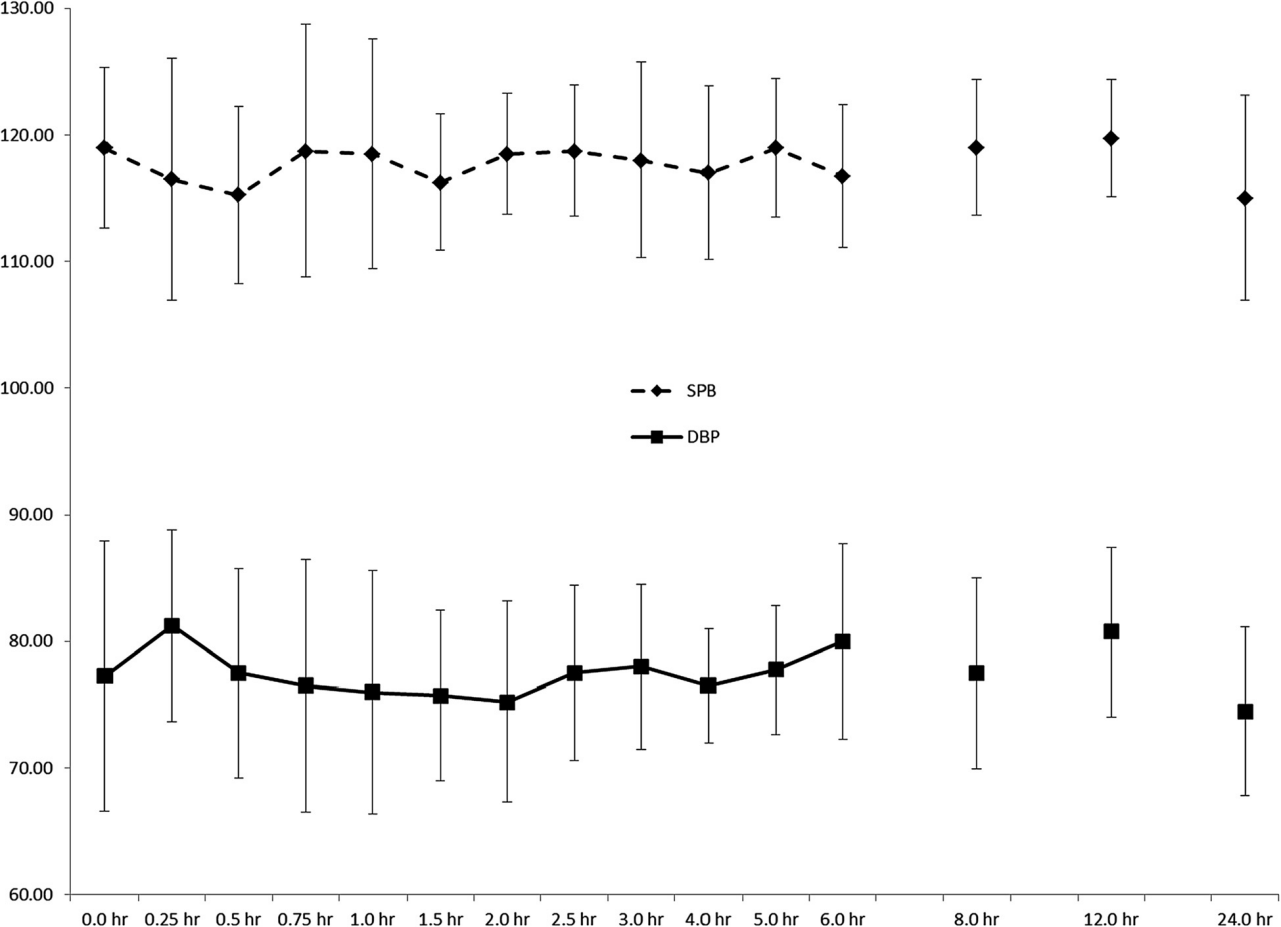
**Blood pressure responses (mm Hg) after oral administration of 25 mg DMAA (n = 7).** Data are mean ± SD. No significant time effects noted (p = 0.271 for diastolic blood pressure and p = 0.722 for systolic blood pressure).

**Figure 6:**
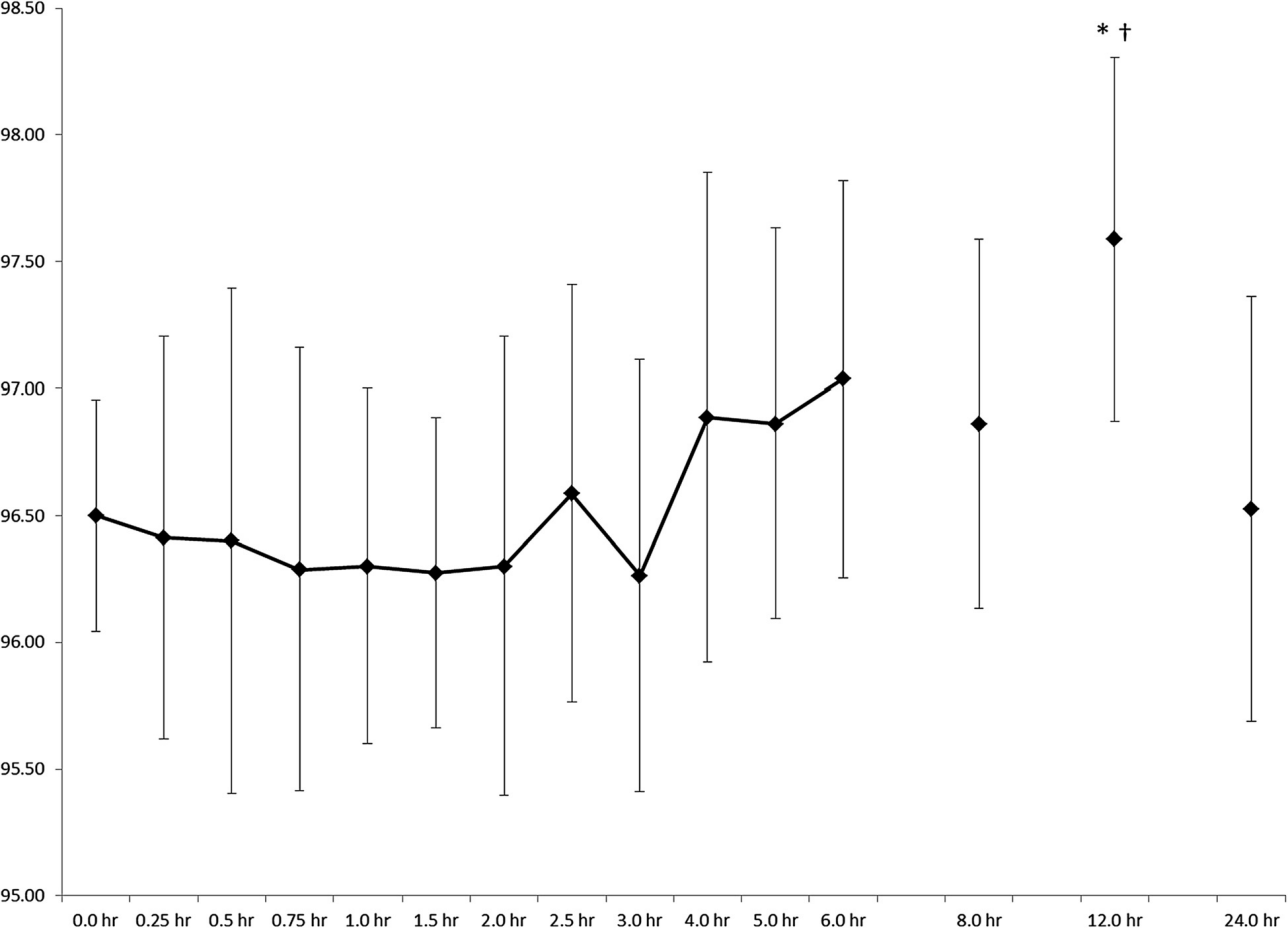
**Temperature (°F) after oral administration of 25 mg DMAA (n = 7). There was a significant time effect (p = 0.001) noted.** * indicates 12-hour greater than 2 hr (p = 0.025). † indicates 12-hour greater than 3-hr (p = 0.009). Data are mean ± SD.

A significant time effect was observed for heart rate (p < 0.000; Figure 4), but no significant pairwise differences were noted. It should be noted that heart rate was slightly elevated at 12 hours post-ingestion (69.1 ± 2.9 BPM) compared to baseline (61.0 ± 3.2 BPM), with a large effect size of 1.9. A significant time effect (p = 0.001) was observed for temperature (Figure 5), with values significantly elevated 12-hours post-ingestion when compared to two hours (p = 0.025, ES = 4.3) and three hours (p = 0.009, ES = 3.4). No changes were seen over time for blood pressure (Figure 6). Systolic blood pressure exhibited a moderate-to-large effect size (0.9) when comparing values at 24 hours post-ingestion (115 ± 3 mm Hg) vs. pre-ingestion (118 ± 3 mm Hg). There was a moderate effect size (0.5) when comparing diastolic blood pressure values at baseline (77 ± 4.4 mmHg) and at 0.25 hr post-ingestion (82 ± 3 mm Hg).

### Pharmacokinetic data

**Figure 7:**
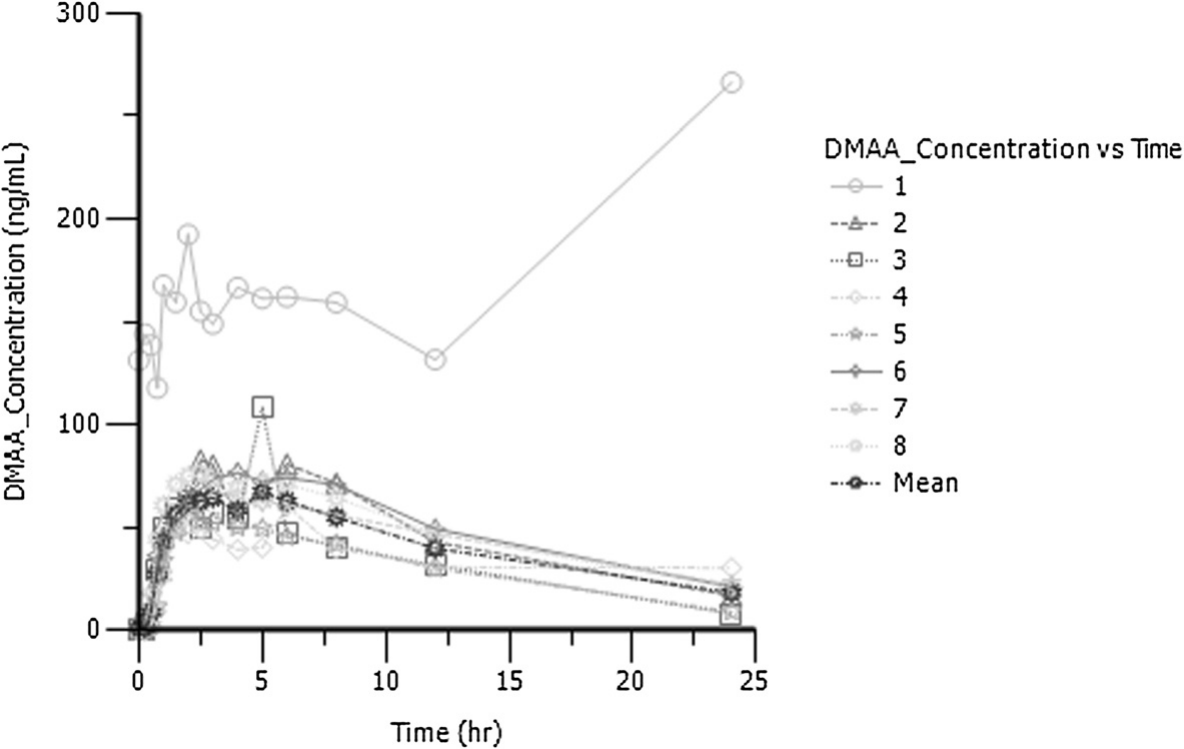
Individual and mean plasma concentration-time profiles after oral administration of 25 mg DMAA.

Each subjects’ pharmacokinetic parameters are shown in Table 2. Values for plasma DMAA of subjects peaked at approximately 70 ng∙mL^-1^. The oral clearance was 20.02 ± 5 L∙hr^-1^, the oral volume of distribution was 236 ± 38 L, and terminal half-life was 8.45 ± 1.9 hr. Lag time varied among participants but averaged approximately 8 minutes (0.14 ± 0.13 hr). Individual and mean DMAA plasma-concentration time profiles are shown in Figure 7.

**Table 2:** Individual pharmacokinetic parameters after oral administration of 25 mg DMAA (n = 7)

**Subject**	**t**_**1/2 **_**(hr)**	**t**_**lag **_**(hr)**	**t**_**max **_**(hr)**	**C**_**max **_**(ng∙mL**^**-1**^**)**	**AUC**_**0-∞ **_**(hr ng∙mL**^**-1**^**)**	**V**_**z**_**/F (L)**	**Cl/F (L∙hr**^**-1**^**)**
2	7.84	0.0	2.5	82.85	1281.88	203.04	17.94
3	6.92	0.25	5.0	108.1	893.23	257.13	25.75
4	6.57	0.25	6.0	60.23	1136.17	191.87	20.24
5	7.13	0.25	2.0	63.14	818.91	289.05	28.09
6	9.29	0.0	4.0	76.68	1456.19	211.73	15.79
7	11.79	0.25	3.0	68.07	1420.82	275.29	16.19
8	9.660	0.0	2.5	76.69	1424.23	223.55	16.15
Mean	8.449	0.143	3.57	76.54	1204.550	235.95	20.02
SD	1.87788	0.134	1.48	16.09	262.89	37.82	5.00
%CV	22.22	93.71	41.46	21.02	21.83	16.03	24.96

## Discussion

The most important finding in this investigation is the relatively low plasma concentrations of DMAA corresponding to the 25 mg oral dose, and the lack of meaningful physiologic effects associated with the single dose. Since data from a standardized and verified dose of DMAA was not previously available, our findings shed light on the possible reason for the adverse outcomes noted in prior case reports citing DMAA use (*i*.*e*., highly abusive dosages of this ingredient) [5,6]. Our data show a consistent pattern of increase across subjects with regards to peak plasma DMAA concentration, with peak values approximately 15–30 times lower than those reported in the case studies—strongly questioning the accuracy of reporting by patients in the case reports[5,6]. It is hypothesized that patients in the case reports may have ingested dosages of DMAA that were approximately 15–30 times higher than what our subjects ingested (*i*.*e*., 375 mg-750 mg). In fact, based on the time course of our peak response data (~5 hours post-ingestion), coupled with the times provided by Gee *et al*.[5,6] for blood sample collection from their patients (*i*.*e*., before or after our noted peak concentration time), it is possible that our “15-30 times higher” estimation is quite low. The conclusions from our data are based on the assumption of linearity of DMAA PK, and that no previous DMAA was ingested by the participants. Also, no assumption of linearity can be made from our data since only one dose was used. The information regarding DMAA dosage as reported by patients in the work of Gee *et al*. [5,6] is not supported by our controlled laboratory analysis involving plasma sample analysis. If these patients did in fact ingest such high dosages of DMAA (as directly indicated in the earlier report of Gee and coworkers, where the subject reportedly ingested 556 mg of DMAA) [6], it should not be surprising that such a blatant abuse resulted in untoward effects. This is particularly true when considering that these patients may have been using other chemicals along with the DMAA (*e*.*g*., alcohol, caffeine, phenethylamine, cannabis). Indeed, the ingestion of other “stimulant-like” substances such as caffeine at a similarly high concentration taken as one dosage (*e*.*g*., 2250 mg-4500 mg; assuming a typical intake of 150 mg) could be highly problematic.

The abnormal response of the one subject in our study cannot be readily explained. This subject did say that he was lightheaded immediately after the catheter placement, but this quickly subsided and was not present at the time of the DMAA administration. Since the subject denied taking DMAA within the 72-hour window preceding the experiment, it remains unclear why these values were so different from the other participants.

Compared to the commonly available stimulant caffeine, DMAA has a longer t_1/2_, in this case 8.4 h vs. 5.4 hr for caffeine [17], as well as a shorter lag time of 0.14 h vs. 0.37 h for caffeine [17]. Previous reports have indicated that DMAA is absorbed over 4–12 hours [13]. It should be noted that caffeine also has interactive effects with oral contraceptives (increasing t_1/2_) and other simultaneously ingested stimulants [17]. Examining the pharmacokinetics of combined DMAA/caffeine ingestion (as is commonly available in supplement formulations) could provide interesting data.

While a significant increase in temperature at 12-hours post-ingestion is noted in our data, the values are still within normal range of 36.1 to 37.8°C, suggesting little meaningful effect is present. The increase in temperature is likely attributable to the fact that subjects were out of the lab and reported back for testing, and that activity associated with leaving and returning to the lab slightly elevated temperature. These data are important to ensure that reports in the lay media of those reportedly taking DMAA suffering heat injury [18] can be contextualized. Further study of DMAA effects on temperature in the context of exercise and heat exposure is warranted.

Even with the significant time effect for heart rate, the grand mean was 64 bpm and the range was 50–88 bpm, well within normal clinical values for healthy young men. Farney *et al*. [9] noted maximum increases of about 7 bpm 90 minutes after ingestion of OxyELITE Pro® (a supplement containing DMAA along with several other ingredients), which is more than the 3 bpm change we noted at the same time period after ingestion of DMAA alone. Our heart rate data are more similar to McCarthy *et al*. [10], showing an increase of 4 bpm at 120 minutes post-ingestion of Jack3d® (another supplement containing DMAA along with other ingredients). It should be noted that McCarthy *et al*. [10] demonstrated an increase of about 4 bpm at 120 min post-ingestion of OxyELITE Pro®, similar to Farney *et al*. [9]. Our data are perhaps best compared with those of Bloomer *et al*. [8] who actually noted a decrease in heart rate of about 4 bpm and 3 bpm with ingestion of 50 mg and 75 mg of DMAA alone, respectively. Since all of these values are within normal ranges, it appears the effects of DMAA on heart rate are indeed minimal.

The lack of time effects for blood pressure is not surprising, and these values are within normal clinical ranges and well below values for hypertension. The systolic blood pressure grand mean was 118 mm Hg, with a range of 96–130 mm Hg. The diastolic blood pressure grand mean was 78 mm Hg, ranging from 60–88 mm Hg. Farney *et al*. [9] noted a significant increase in systolic blood pressure at 60, 90, and 120 post-ingestion of OxyELITE Pro®, but not Jack3d®. However, their maximum values were very similar for the two substances, and all of their values are similar to ours, aside from the greater baseline values for our study. McCarthy *et al*. [10] also had a significant time effect for OxyELITE Pro® on systolic blood pressure, and again their maximum values are similar to those herein. Study of non-proprietary concentrations of DMAA [8] demonstrated somewhat greater blood pressure effects for 50 and 75 mg (~8 and 12 mm Hg) than we noted for our 25 mg dose. Of the aforementioned studies, only Bloomer *et al*. [8] reported a significant increase in diastolic blood pressure, at both 50 and 75 mg doses, and at time points 30, 60, and 90 minutes post-ingestion.

## Conclusions

We report for the first time the pharmacokinetic profile of oral DMAA. Based on our data, it appears that the concern over adverse health-related effects of DMAA is specific to the dosage ingested by the individual. When ingested at recommended doses (*e*.*g*., 25 mg), our data indicate minimal to no change in heart rate, blood pressure, or body temperature, and no adverse effects were noted. We also note a consistent pattern of increase across subjects concerning peak DMAA concentration, with mean peak values being <77 ng∙mL^-1^. This is approximately 15–30 times lower than plasma values reported by other investigators citing adverse outcomes following DMAA use. However, due to the case-study format of some of these adverse events, one cannot ignore possible drug interaction, errors in bioanalytical methods, differing bioavailability, or variability in exposure that might make this comparison difficult. Interpretation of our data would lead one to hypothesize that the adverse outcomes associated with DMAA use are simply due to the blatant abuse of this ingredient. Future research on DMAA may consider pharmacokinetic characterization of each individual diastereoisomer [14,15].

## Competing interests

BKS, KGH and RJB have received research funding from USPlabs, including this study. These contracts paid for direct and indirect costs, as well as salary. USPlabs has also paid for the article-processing fee. This study was funded by USPlabs, who was consulted in the design of the study.

## Authors’ contributions

BKS and KGH were involved in the conception and design of the study, acquisition of data, analysis of data, and manuscript preparation. RJB was involved in the conception and design of the study, and manuscript preparation. CSP and CRY were involved in analysis of data and assistance with manuscript preparation. All authors read and approved the final manuscript.

## Pre-publication history

The pre-publication history for this paper can be accessed here:

http://www.biomedcentral.com/2050-6511/14/52/prepub
